# What Does It Take to Become an Academic Plastic Surgeon in Canada: Hiring Trends Over the Last 50 Years

**DOI:** 10.1177/22925503211011974

**Published:** 2021-05-17

**Authors:** Andrea E. Copeland, Daniel E. Axelrod, Chloe R. Wong, Janna L. Malone, Lucas Gallo, Ronen Avram, Brett T. Phillips, Christopher J. Coroneos

**Affiliations:** 1 Division of Plastic Surgery, Department of Surgery, McMaster University, Hamilton, Ontario, Canada; 2 Division of Orthopaedic Surgery, Department of Surgery, McMaster University, Hamilton, Ontario, Canada; 3 Michael G. DeGroote School of Medicine, McMaster University, Hamilton, Ontario, Canada; 4 Division of Plastic, Maxillofacial, and Oral Surgery, Duke Department of Surgery, Duke University School of Medicine, Durham, NC, USA

**Keywords:** employment, surgery, plastic, gender, hiring

## Abstract

**Objective:** Academic plastic surgery positions have become highly competitive secondary to delayed retirement, stagnant hospital funding, and an increasing number of plastic surgery graduates. Little information is available to help residents navigate this challenging landscape. Our objectives were to evaluate the training backgrounds of all Canadian academic plastic surgeons and to develop recommendations for residents interested in an academic career. **Methods:** All Canadian academic plastic surgeons were included. Training histories were obtained from institutions’ websites. Surgeons were subsequently emailed to confirm this information and complete missing details. Multivariate regressions were designed to analyze the effect of gender and FRCSC year on graduate and fellowship training and time to first academic position. **Results:** Training information was available for 196 surgeons (22% female), with a 56% email response rate; 91% of surgeons completed residency in Canada; 94% completed fellowship training, while 43% held graduate degrees; 74% were employed where they previously trained. Female gender significantly lengthened the time from graduation to first academic job, despite equal qualification. Younger surgeons were more likely to hold graduate degrees (*P* < .01). **Conclusions:** We identified objective data that correlate with being hired at an academic centre, including training at the same institution, obtaining a graduate degree during residency, and pursuing fellowship training. In addition, we demonstrated that women take significantly longer to acquire academic positions (*P* < .01), despite equal qualification. Trainees should consider these patterns when planning their careers. Future research should explore gender-based discrepancies in hiring practices.

## Résumé

**Objectif:** Les postes universitaires en chirurgie plastique sont hautement convoités en raison des retraites reportées, du gel du financement des hôpitaux et d’un nombre grandissant de diplômés en chirurgie plastique. Il y a peu d’information visant à aider les résidents à s’y retrouver dans ce contexte difficile. Nos objectifs consistaient à évaluer la formation de tous les chirurgiens plasticiens universitaires au Canada et de mettre au point des recommandations à l’intention des résidents souhaitant mener une carrière universitaire. **Méthodologie:** Tous les chirurgiens plasticiens universitaires du Canada ont été inclus. L’historique de formation a été obtenue auprès des sites Web des établissements. On a ensuite fait parvenir aux chirurgiens un courriel visant à confirmer ces renseignements et à compléter l’information manquante. Des régressions multifactorielles ont été conçues pour analyser l’effet du sexe et de l’année d’obtention du titre FRCSC sur l’accès à la formation aux cycles supérieurs et l’octroi des bourses de formation, ainsi que sur le délai précédant le moment où les chirurgiens plasticiens décrochent leur premier poste universitaire. **Résultats:** Les renseignements sur la formation étaient disponibles pour 196 chirurgiens (dont 22 % de sexe féminin), et le taux de réponse par courriel a été de 56 %. Quatre-vingt-onze pour cent des chirurgiens ont terminé leur résidence au Canada. Quatre-vingt-quatorze pour cent des chirurgiens avaient reçu une bourse de formation, tandis que quarante-trois pour cent détenaient des diplômes d’études supérieures. Soixante-quatorze pour cent des chirurgiens sont devenus des employés de l’établissement où ils avaient déjà suivi une formation. À compétences égales, les chirurgiens de sexe féminin tardaient beaucoup plus à décrocher un premier emploi universitaire après l’obtention de leur diplôme que ceux de sexe masculin. Plus les chirurgiens étaient jeunes, plus ils étaient susceptibles de détenir un diplôme d’études supérieures (*p* < 0,01). **Conclusions:** Nous avons obtenu des données objectives présentant une corrélation avec l’embauche dans un centre universitaire, y compris une formation au même établissement, l’obtention d’un diplôme d’études supérieures durant la résidence et le maintien d’une bourse de formation. En outre, nous avons montré que même à compétences égales, les femmes prennent beaucoup plus de temps à décrocher des postes universitaires (*p* < 0,01). Les étudiants doivent tenir compte de ces profils dans leur planification de carrière. Des recherches futures devront explorer les écarts entre les sexes pour ce qui est des pratiques d’embauche.

## Introduction

Academic plastics surgeons typically provide specialized clinical care and perform research while simultaneously training the next cohort of surgeons. In a recent survey, 38% of Canadian plastic surgery residents were interested in academic practice.^1^ In the same study, more than half of Canadian plastic surgeons worked in either a purely academic (29%) or a mixed practice (24%).^1^

Despite attempts at workforce planning, there remains a disconnect between the number of residents being trained and the demand for new plastic surgeons.^1,2^ In the last 10 years, there has been an increase in the number of graduating plastic surgery residents.^3^ Unfortunately, the job market has not accommodated for this, with fewer surgeons retiring and no means to increase hospital resources to accommodate more plastic surgeons.^3^

Beyond the imbalance in surgeon supply and demand, there exists a discrepancy in health care funding and patient needs; as the health care needs of patients continue to increase, hospital funding in our single payer, publicly funded health care system has been paradoxically reduced.^4^ Necessary resources like operating room time and inpatient beds are frequently cut.^4^ This reality was reflected in a 2007 survey^5^ where 75% of surgeons indicated there were not enough plastic surgeons in their hospital/community, yet only 25% felt their government/hospital would provide the necessary resources to accommodate another plastic surgeon. A more recent workforce analysis^6^ demonstrated improvement (59% and 36%, respectively); however, the discrepancy between surgeon perception of hiring needs and hospital funding remains. Since workforce planning tends to consider only societal health needs but not the availability of practice resources,^4^ the competition plastic surgeons face in finding a job is unlikely to change while health care budgets remain low.

For existing plastic surgery residents, it can be challenging to navigate this landscape. Trainees are given limited information regarding the relative benefit of fellowships, graduate degrees, and the location of their training.^7,8^ This issue is magnified, particularly within the academic plastic surgery community, as research productivity and graduate degrees are expected for employment.^9,10^

Moreover, there is little available literature on this matter for residents to review when planning their surgical careers. Previous American studies have identified factors that may predict future academic employment, including a greater number of publications before and during residency, stronger perceived mentorship during residency, and completion of fellowship training.^11^ Unfortunately, the results from American studies cannot be directly applied to Canadian practices given the differences in medical, legal, and economic circumstances.^12^

There are 2 relevant Canadian studies. In 1998, Fish and McKee^13^ surveyed program directors and found that while no programs required a Master’s or doctorate (PhD) degree for new academic plastic surgery appointments, 35% of respondents indicated that it was preferred. In addition, 100% of program directors felt that a specific area of clinical interest was very important, although fellowship training was not specifically targeted in the survey. A more recent survey^3^ found that professional reputation, number of publications, and a letter from the program director were important factors to consider when hiring a new surgeon.

In both articles, the authors developed opinion-based surveys, asking plastic surgeons in leadership roles what qualities *they* felt were important. There have been no Canadian studies that analyze relevant demographics and training backgrounds of plastic surgeons currently in practice in Canadian academic centres. Moreover, the existing literature reflects a snapshot at a given time rather than assessing hiring trends and how academic practices have changed over time. Finally, although gender disparities have been studied in academic medicine and other surgical specialties,^14-21^ the extent to which gender impacts hiring practices in plastic surgery has not been well defined.

The primary objective of this study is to analyze the training backgrounds of academic plastic surgeons in Canada, including fellowships, graduate degrees, and location of training. Based on these findings, we will develop recommendations for residents interested in an academic career. Secondary objectives include identifying temporal trends and gender disparities in the employment of Canadian plastic surgeons.

## Methods

This study was exempt from research ethics board review at our institution. Participating surgeons were informed that survey results were anonymous and that results would be reported in aggregate. Academic surgeons were defined as those affiliated with the plastic surgery division at each respective institution. Our list of current (July 24, 2019) academic plastic surgeons (n = 199) was obtained through each academic institution and by contacting program administrators. Surgeon information was collected by a single reviewer using each institution’s public websites, online faculty profiles from private practice websites, and LinkedIn. The following information was obtained: gender, residency training program, year of Canadian board certification (FRCSC), subspecialty fellowship completion, advanced graduate degrees obtained (completed before, during, or after residency), and the year and location of first academic job (excluding locums). All respondents were confirmed to hold a full academic appointment, but rank at hire was not collected. Following our collection, each surgeon was sent their own information electronically to confirm its accuracy and complete missing information. Those who did not respond were contacted again electronically after 2 weeks. In the case of a disagreement between the information collected online and the surgeon’s response, the latter was used for analysis.

Time to job was calculated as year of FRCSC to year of first academic job. FRCSC year was used as a surrogate for age. Surgeons were divided into an “older” group if their FRCSC was obtained before the year 2000 and “younger” if in the year 2000 or later. The year 2000 was used as this approximately divided the cohort in half and represented the mean FRCSC of all included surgeons. Clinical fellowships were subdivided into microsurgery, hand, craniofacial, burn, pediatric, aesthetic, and other. Primary comparison of continuous demographic data was assessed with unpaired Student *t* tests. Categorical data were analyzed through χ^2^ tests, utilizing a Yates correction when categories did not have enough subjects. Multivariate analysis was performed to assess whether FRCSC year and gender predicted presence/absence of graduate degree, presence/absence of fellowship training, mean number of fellowships, and time from FRCSC to first job. Covariates were identified a priori. Lastly, a time to event analysis, with time to job as the dependent variable, was undertaken to generate hazard ratios. R (Open Access, Version 3.6.1) was utilized for all statistical analyses.

## Results

**Figure 1.**
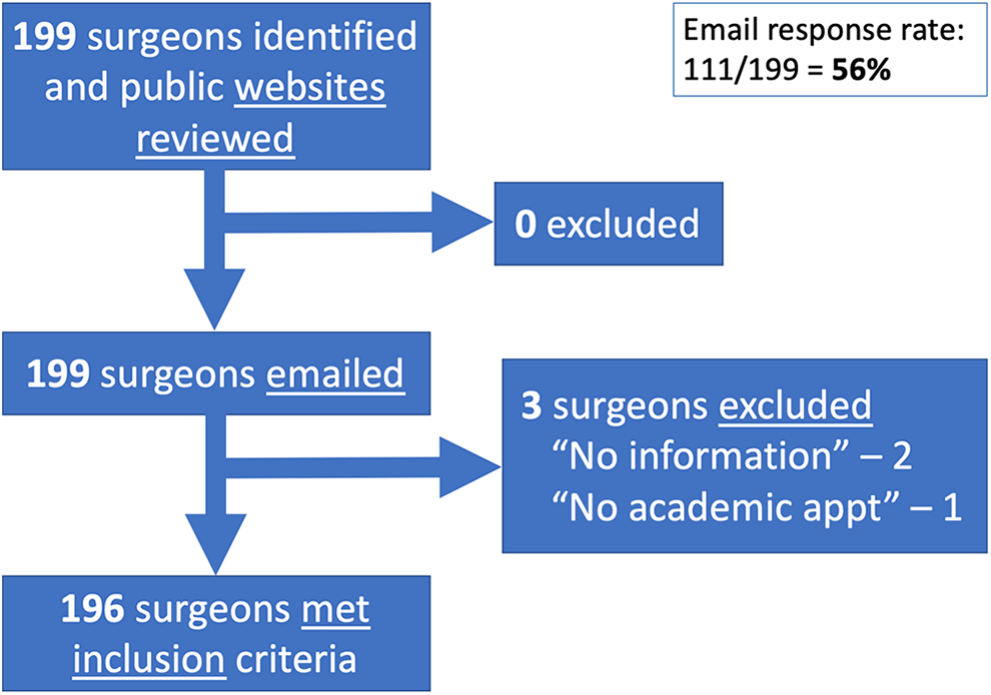
Flow diagram demonstrating inclusion and exclusion.

Information was collected for 196 of 199 identified surgeons. Two were excluded because no information was available and one was excluded for not actually having held an academic appointment (Figure 1). Review responses were received from 56% (111) of surgeons. Among the responders whose information had all been available online (n = 26), 22 confirmed the data without making any corrections and 4 made corrections. Among the responders who were missing online data (n = 85), 83 confirmed the existing data and filled in the missing details, while 2 made corrections to existing data while also filling in missing data. The κ agreement statistic was 0.845, suggesting almost perfect agreement.

### Characteristics of Academic Plastic Surgeons in Canada

Of the 196 surgeons, 22% were female. Mean year of board certification (FRCSC) for all surgeons was 2000 and median was 2002. These data are presented in Table 1. Female surgeons were significantly younger based on mean year of board certification (2007 vs 1998, *P* < .0001; Table 3).

**Table 1.** Demographic and Training Pathway Data.

Surgeon Demographic and Training Characteristics	N (%)
Female	43 (21.9)
Male	153 (78.1)
FRCSC 2000 or later (“younger”)	113 (57.5)
FRCSC before 2000 (“older”)	83 (42.3)
Canadian Residency	175 (90.7)
International Residency	18 (9.3)
Graduate degree	84 (43.3)
Master’s	66 (34.0)
Doctorate	18 (9.3)
Before Residency	22 (11.6)
During Residency	33 (17.5)
After Residency	25 (13.2)
Fellowship	181 (93.8)
One	106 (54.9)
Two or more	75 (38.9)
Canadian	91 (47.4)
International	129 (67.2)
Completed a Fellowship in Same City as Residency	42 (23.3)
Hired in Same City as Residency alone	101 (55.5)
Hired in Same City as Fellowship alone	12 (6.6)
Hired in Same City as Residency and Fellowship	20 (11.0)
Hired in Different City from Residency/Fellowship	49 (26.0)

FRCSC, Fellow of The Royal College of Physicians of Canada.

### Training Pathways

**Figure 2.**
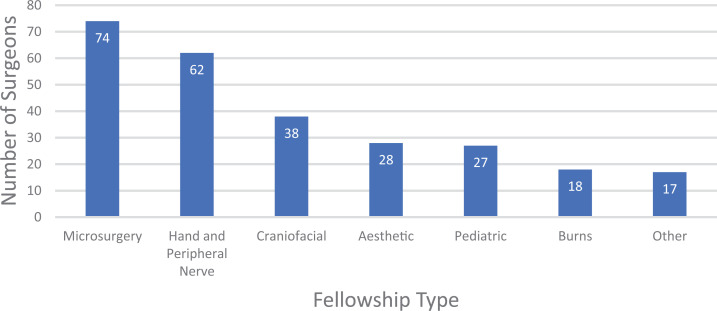
Fellowship subspecialty distribution.

The majority of surgeons completed their plastic surgery residency training in Canada (91%). Overall, 43% of surgeons held a graduate degree with 77% of these being Master’s degrees, and the most common time of completion being integrated during residency (41%, n = 33). Fellowship training was completed by 94%, with the mean number of fellowships being 1.4; 67% completed international fellowships and 47% completed Canadian fellowships; 23% remained at the same institution for fellowship following their residency training. The most common subspecialty training was microsurgery (28%), followed by hand surgery (24%). Complete fellowship subspecialty distribution is shown in Figure 2. The overall mean number of years from FRCSC to first academic job, excluding locums, was 1.49 years. The majority (67%) of surgeons were hired in the same city in which they completed their residency training. An additional 7% of surgeons were hired in the city they completed their fellowship, without having completed their residency there. Therefore, overall 74% of surgeons were hired at an institution at which they were trainees. Complete training pathway data are present in Table 1.

### Trends in Hiring Practices

Regression analyses demonstrate that when controlling for gender, younger surgeons were significantly more likely than older surgeons to hold a graduate degree (49% vs 36%, respectively, *P* = .007). There was no difference between younger and older surgeons in completing fellowship training (96% vs 92%, *P* = .26), mean number of fellowships (1.51 vs 1.34, *P* = .138), or time to first job (1.38 vs 1.41, *P* = .45). All significant relationships were maintained when age was considered as a continuous variable. Temporal trend data are presented in Table 2.

**Table 2.** Temporal Trends in Training Pathways.

Surgeon Demographic and Training Characteristics	Older^a^	Younger^b^	*P* value
Females, n (%)	9 (10.7)	34 (30.4)	<.001
Surgeons with graduate degree, n (%)	30 (35.7)	54 (49.1)	.007
Surgeons with fellowship training, n (%)	77 (91.7)	107 (95.5)	.260
Number of fellowships, mean	1.34	1.51	.138
Time from FRCSC to first academic job in years, mean	1.41	1.38	.450

^a^ FRCSC before 2000.

^b^ FRCSC 2000 or later

FRCSC, Fellow of The Royal College of Physicians of Canada.

### Gender Disparities in Hiring Practices

**Figure 3.**
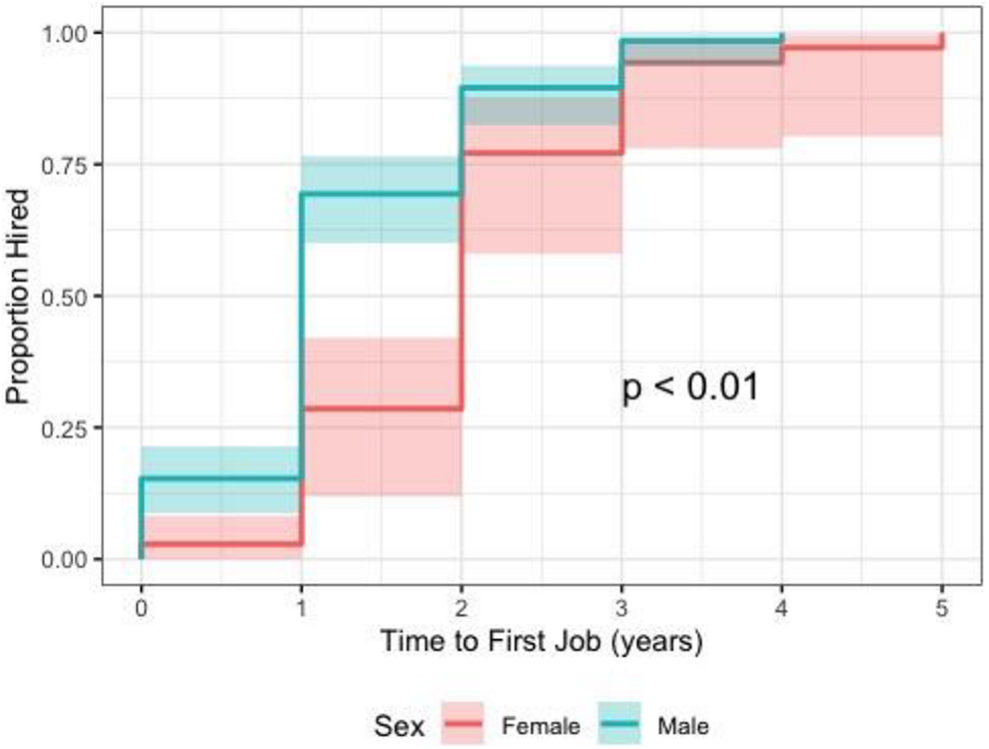
Time to event analysis for time to first academic job, stratified by gender.

Overall, female surgeons took significantly longer to be hired after their FRCSC certification than male surgeons (2.04 vs 1.22 years, *P* = .003). This effect was strengthened in multivariable analysis when controlling for potential confounders: FRCSC year, graduate degrees, and fellowships (*P* < .0001). In time to event analysis, females had a significantly lower chance of being hired at each one-year time interval (hazard ratio = 1.8, 95% CI: 1.3-2.7, *P* = .002; Figure 3). Female surgeons were also more likely to have graduate degrees (56% vs 40%), but this did not reach significance in multivariate analysis (*P* = .166) when controlling for age. There was no gender difference in presence/absence of fellowship (95% vs 93%, *P* = .96) but the mean number of fellowships trended toward significance in regression analysis with females completing 1.53 and males completing 1.38 fellowships (*P* = .06). Gender differences in hiring are presented in Table 3.

**Table 3.** Gender Differences in Training Pathways.

Surgeon Demographic and Training Characteristics	Female	Male	*P* value
FRCSC year, mean (SD)	2007 (9)	1998 (12)	<.001
Surgeons with graduate degree, N (%)	23 (56)	61 (40)	.166
Surgeons with fellowship training, N (%)	40 (95)	143 (93)	.960
Number of fellowships, mean (SD)	1.53 (0.82)	1.38 (0.72)	.060
Time from FRCSC to first academic job in years, mean (SD)	2.04 (0.96)	1.22 (0.80)	<.001

FRCSC, Fellow of The Royal College of Physicians of Canada.

## Discussion

This is the first analysis of academic backgrounds of all Canadian plastic surgeons practicing at academic centres. We identified objective data that correlate with being hired at an academic centre. For trainees interested in an academic career, we recommend obtaining a graduate degree during residency, pursuing 1 to 2 fellowships, and training in the city where they would like to eventually be hired. In addition, we were able to analyze temporal trends given the wide age range of current academic plastic surgeons. Finally, we demonstrated that female gender significantly lengthens the time from graduation to first academic job, even after controlling for any other training factor. This gender disparity in time to job acquisition has not previously been reported in the Canadian literature.

Given the competitive job market in plastic surgery,^3^ residents are understandably concerned about their post-residency future. A recent study by Morzycki et al^6^ showed that “89% of plastic surgery residents plan to pursue additional post-residency training, with 70% stating that the current job market is contributing to their decision.” Unfortunately, there is little literature available to guide residents as they navigate this challenging landscape, particularly for graduates who wish to pursue academic positions. They therefore rely on mentorship, which is neither formalized in all residency programs nor consistent for all residents.^22-25^

In our study, the majority (94%) of Canadian academic plastic surgeons completed fellowship training, with surgeons completing an average of 1.41 fellowships. This corresponds with our finding that mean time to job was 1.49 years, given that the majority of fellowships in plastic surgery are one year in duration.^26^ Mean number of fellowships was not affected by FRCSC year, implying the long-standing importance of a subspecialty focus in Canadian academic plastic surgery. However, there is a potential ceiling effect given the high proportion of both older and younger graduates with fellowship training, suggesting little potential for a temporal difference to be shown statistically. Graduate study, compared to fellowship training, is less common among academic plastic surgeons, with just under half (43%) of surgeons holding a graduate degree. This, however, may be changing, as significantly more “younger” compared to “older” surgeons hold graduate degrees, which may reflect an increase in job competitiveness and a need for graduates to distinguish themselves.

In contrast to our findings, an American study published in 2014^27^ found that only 58% of surgeons completed a subspecialty fellowship and 11% held graduate degrees (compared to 94% and 43% in our study, respectively). This marked difference suggests that Canadian academic surgery positions are more competitive, which may be largely due to a relatively fewer number of academic centres in Canada. It may also be due to different educational opportunities and importance placed on academics while training. Finally, differences in practice profiles may be a contributing factor. The majority of Canadian academic plastic surgeons (74%) have a reconstructive practice^12^ compared to only 50% of Americans.^28^ American surgeons spend more time in offsite private offices and surgical centres, leading to less competition for resources.^12^ We hypothesize that these differences make it easier for graduating plastic surgery residents to be hired into academic practice in the United States, as resources do not impede new hires.

In 2019, Oxley and Lotto surveyed practicing plastic surgeons about factors that influence them when selecting new colleagues.^3^ When academic and community surgeons were separated, they found that academic centres were significantly more likely to value graduate degrees, number of publications, and location and duration of fellowship training. In another recent survey by Morzycki et al,^6^ 89% of Canadian plastic surgery residents plan to pursue additional training.

Perhaps one of the most interesting findings is that the majority (67%) of surgeons were first hired in the same city in which they completed their residency. Although a causal relationship cannot be proven, internal hiring is certainly logical in that the applicants’ ability and skills are known, and the applicant already understands the institution’s environment.^29^ Of the 121 surgeons who were hired in their residency city, 101 completed fellowship elsewhere. Completing subspecialty training elsewhere is advantageous since trainees acquire new clinical and academic expertise to return to their home institution.

In contrast to our findings, the survey of practicing surgeons by Oxley and Lotto^3^ showed that among the *least* important factors when hiring a new surgeon were the candidate having previously trained with the group, completing residency at the nearest training group, and attending a local university for their medical school training. Since this study reflected surgeons’ opinions on desired traits, rather than objective findings, this apparent discrepancy may reflect the *ideal* versus *observed* behaviour when selecting a candidate. Ideally, there is no bias toward “hiring your own” but in reality, the overwhelming familiarity of the candidate among all surgeons on the hiring committee cannot be ignored, whether consciously or not.

Finally, gender disparities in surgery have been well documented^16,17,21^ but are not well defined in the plastic surgery literature. In our study, women were underrepresented (22%). It is encouraging though that women were significantly more likely to have a later FRCSC, indicating that female representation is improving. Our numbers were similar to a recent Canadian study^1^ that showed 22% of *all* Canadian plastic surgeons are female, which suggests that female representation is about 22% in both academic and non-academic centres. In the United States, only 14% of plastic surgeons are female.^27,30^ Both external and internal explanations have been postulated for female under-representation, including lack of female mentorship, low compensation compared with male colleagues, difficulties in professional advancement despite equal qualification, differences in family responsibilities which can contribute to poor work–life balance and professional dissatisfaction, and gender differences in negotiation and communication skills.^31^

Our study found that despite being at least equally, and possibly more qualified, female plastic surgeons took *significantly longer to be hired* onto their first academic job. In the 2019 survey by Oxley et al, greater time since residency training was perceived as a negative factor when considering applicants for academic jobs; that is, fresher/more recently trained graduates were preferred.^3^ This, combined with our results, suggests that women are significantly disadvantaged when applying for academic jobs.

More research is needed to characterize the reason female plastic surgeons are taking longer to advance to the next stage of their career. One explanation is that women delay their career for pregnancy and childrearing. Several studies have shown that female physicians bear a greater burden of domestic and parenting responsibility than male physicians.^32,33^ However, whether this leads to a delay in job acquisition is less clear. In fact, female plastic surgeons have been shown to be more likely to be unmarried, marry later, and delay having children compared to their male counterparts.^30,34,35^ Furthermore, increasingly more female surgical residents are choosing to have children during residency,^36^ and taking time off *during* residency would not affect time from FRCSC to first academic job. Another explanation is that female physicians are more restricted by location when applying for jobs since they are more likely than male physicians to have spouses that are employed full time.^33^ Having fewer job options available may extend the time to full employment. Finally, there may be systemic gender bias in hiring processes. Regardless of the reason, a delay in job acquisition represents lost time fully employed.

This study has limitations. Firstly, it relied largely on public information listed on institution and networking websites, which may not have been complete or up to date. We improved the quality of our data by directly emailing all surgeons and asking them to confirm existing data and/or provide missing data. Our response rate (56%) was very good by conventional survey metrics. Second, certain information was not available online, including fellowship duration and timing of graduate degrees completed after residency. A fellowship that is not one year in duration or a graduate degree completed between FRCSC and first job would both influence “time to job” and were not accounted for. However, these factors would not be influenced by gender and thus should not affect our conclusions regarding gender bias. Third, by limiting the study population to academic plastic surgeons, we were not able to generate a comparison group of non-academic surgeons. Therefore, while we can draw conclusions regarding the criteria needed for an academic position, we cannot determine whether these criteria are important for non-academic jobs. Fourth, our study does not capture surgeons who were hired but have since left academia. These surgeons are more likely to fall into the FRCSC before 2000 cohort and therefore this may have affected our analysis of temporal trends. Fifth, additional factors such as personal and professional reputation, the role of the interview, and mentorship, are all influential in academic hiring.^3,22-25^ This study only assessed training background, gender, and temporal relationships. Finally, reasons for gender disparities in time to job acquisition were not addressed and should be explored in future research.

## Conclusion

Our study summarizes the training backgrounds of Canadian academic plastic surgeons and demonstrates the importance of fellowship training and graduate degrees in the attainment of an academic job, as well as location of residency program. It also demonstrates that women are at a disadvantage when applying for academic positions with a longer time to being hired despite equal and possibly greater qualification. Trainees interested in a career in academia should consider obtaining graduate degrees and additional fellowship training. They should also strongly consider completing their residency training in the city they would like to eventually be hired. Future research should focus on identifying reasons for gender-based discrepancies in hiring practices.
